# Mining comorbidities of opioid use disorder from FDA adverse event reporting system and patient electronic health records

**DOI:** 10.1186/s12911-022-01869-8

**Published:** 2022-06-16

**Authors:** Yiheng Pan, Rong Xu

**Affiliations:** Case Western Reserve University, Cleveland, OH USA

**Keywords:** Opioid use disorder, Biomedical informatics, Data mining, Statistical analysis, Network analysis

## Abstract

**Background:**

Opioid use disorder (OUD) has become an urgent health problem. People with OUD often experience comorbid medical conditions. Systematical approaches to identifying co-occurring conditions of OUD can facilitate a deeper understanding of OUD mechanisms and drug discovery. This study presents an integrated approach combining data mining, network construction and ranking, and hypothesis-driven case–control studies using patient electronic health records (EHRs).

**Methods:**

First, we mined comorbidities from the US Food and Drug Administration Adverse Event Reporting System (FAERS) of 12 million unique case reports using frequent pattern-growth algorithm. The performance of OUD comorbidity mining was measured by precision and recall using manually curated known OUD comorbidities. We then constructed a disease comorbidity network using mined association rules and further prioritized OUD comorbidities. Last, novel OUD comorbidities were independently tested using EHRs of 75 million unique patients.

**Results:**

The OUD comorbidities from association rules mining achieves a precision of 38.7% and a recall of 78.2 Based on the mined rules, the global DCN was constructed with 1916 nodes and 32,175 edges. The network-based OUD ranking result shows that 43 of 55 known OUD comorbidities were in the first decile with a precision of 78.2%. Hypothyroidism and type 2 diabetes were two top-ranked novel OUD comorbidities identified by data mining and network ranking algorithms. Based on EHR-based case–control studies, we showed that patients with OUD had significantly increased risk for hyperthyroidism (AOR = 1.46, 95% CI 1.43–1.49, *p* value < 0.001), hypothyroidism (AOR = 1.45, 95% CI 1.42–1.48, *p* value < 0.001), type 2-diabetes (AOR = 1.28, 95% CI 1.26–1.29, *p* value < 0.001), compared with individuals without OUD.

**Conclusion:**

Our study developed an integrated approach for identifying and validating novel OUD comorbidities from health records of 87 million unique patients (12 million for discovery and 75 million for validation), which can offer new opportunities for OUD mechanism understanding, drug discovery, and multi-component service delivery for co-occurring medical conditions among patients with OUD.

## Background

Opioid is commonly used to relieve pain. However, abuse of opioids is a major public health crisis. An estimated total 450,000 people died from opioid overdose 1999–2018 [1]. Recent research has demonstrated the high rates of co-occurring conditions of opioid use disorder (OUD) [2]. Among the patients with OUD, 27% of patients have serious mental diseases, 64% have any mental diseases, and around 11% to 26% have alcohol use disorder or any other substance use disorders [3]. To optimize multi-component service delivery for co-occurring medical conditions associated with the opioid crisis, the Help to End Addiction Long-term (HEAL) Multidisciplinary Working Group (MDWG) sought research to study the effectiveness of components in these complex patient population in August 2019 and in March 2020 again [4]. Considerable research has been established to study the known comorbidities, such as mental disorders among OUD patients [5]. However, contradictory findings and unclear associations between OUD and other co-occurring health problems still exist, such as type 2 diabetes [6]. Identifying and independently validating OUD comorbidities will provide valuable knowledge to understand mechanisms underlying OUD, develop drug treatment [7], and deliver effective intervention in multi-component service delivery for OUD patients with these co-occurring health problems.

The FDA Adverse Event Reporting System (FAERS) is a public database containing voluntary adverse event reports, medication error reports, and product quality complaints that were submitted to FDA directly from manufacturers, consumers, and clinical professionals. It is documented as quarterly updated data files that include patients’ indication, medication, adverse events, and demographics information [8]. The case report in indication data file records the drugs and diseases for an individual, which can be used to identify disease comorbidities. We previously constructed diseases comorbidity networks (DCNs) for colorectal cancer [9] and Alzheimer’s disease [10] from FAERS using association rule mining and demonstrated that these DCNs were correlated with disease treatment, disease semantic similarity, and disease genetics [11], In this study, we aimed to identify novel OUD comorbidities from FAERS. We validated top-ranked novel OUD comorbidities with de-identified population-level electronic health records (EHRs) of 75 million unique patients available through IBM Watson Explorys platform [12]. We have used the Explorys EHR database and the cloud‐based Explorys Cohort Discovery informatics tools for health outcomes research [13–16] and drug discovery [17–19].

In summary, we discovered novel OUD comorbidities from FAERS of 12 million patients by combining data mining and network-based ranking techniques and independently validated the novel findings with EHRs of 75 million patients. To the best of our knowledge, our study represents the first effort towards large-scale mining and independently validating OUD comorbidities. Novel OUD comorbidity findings from our study will extend the understanding of mechanisms of OUD and provide new opportunities for identifying novel OUD medication and for optimizing multi-component service delivery to the OUD cohort with co-occurring medical conditions.

## Methods

**Fig. 1:**
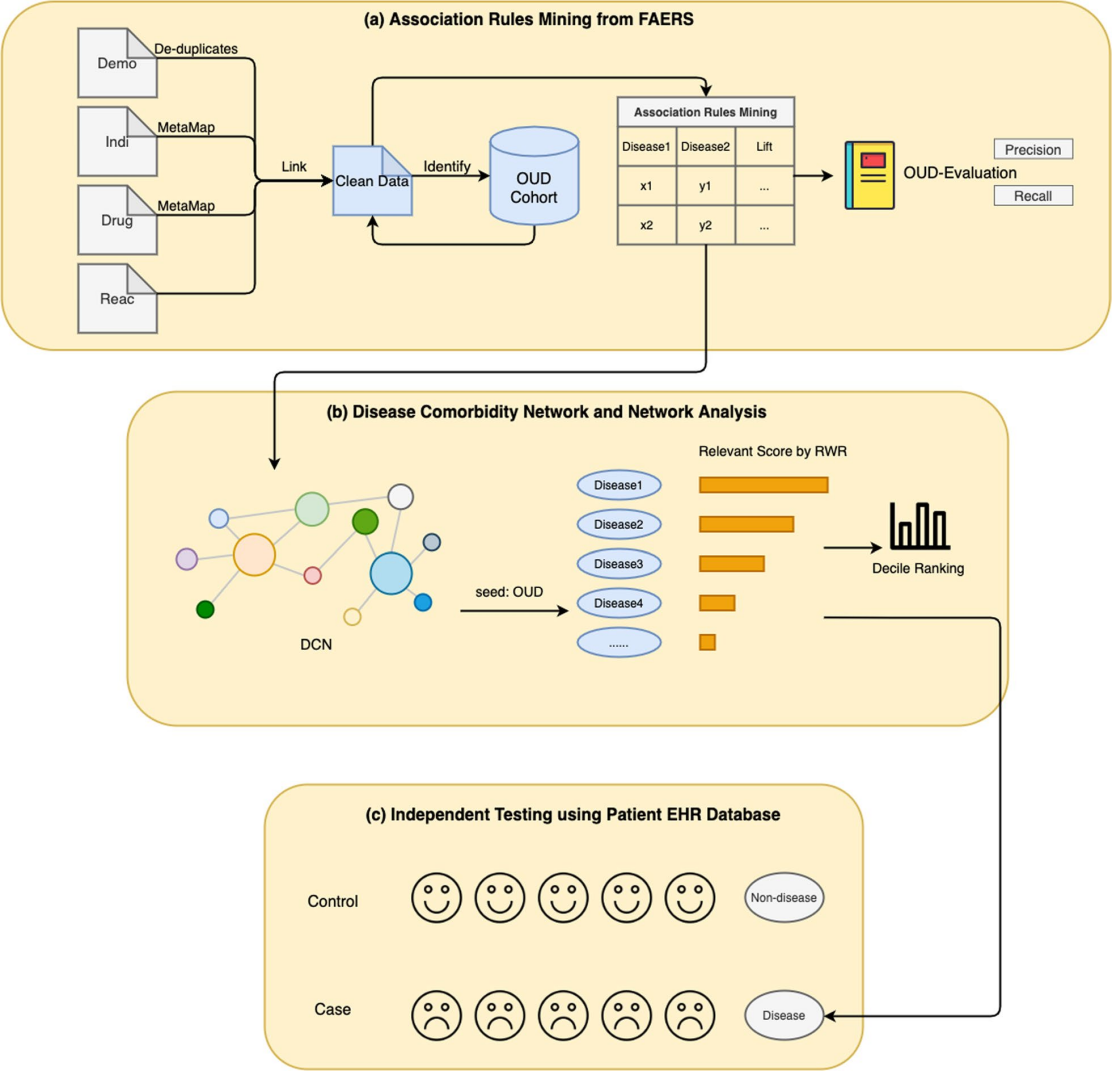
Workflow of our study. OUD: opioid use disorder; RWR: random walk with restart; HER: electronic health record

The overview of our study is shown in Fig. 1. (1) We used association rule mining approach to obtain frequent patterns from processed FAERS data files. (2) We constructed disease comorbidity network (DCN) based on these mined rules and prioritized OUD comorbidities using OUD as the seed. (3) Top ranked novel OUD comorbidities were independently validated using patient EHR database.

### Association rules mining from FAERS

#### Data processing

We downloaded data from FDA Adverse Event Reporting System official website [8], which includes demographics, administration, medications, reaction, and outcomes between 2004 first quarter and 2020 fourth quarter. To keep consistency, we first unified the LAERS file formats into FAERS format. The redundant records were de-duplicated from 14,526,397 to 12,194,096 by only keeping the latest demographics record for each case [20]. We recognized the indication preferred terms and drug names from free text into UMLS [21] standard formats using Metamap (2020 version) [22]. The original drug name, indication term and adverse event term are in mixed structured and free-text short strings in three files, which reflected the observations and the reporters’ opinions. 12,872 of 18,229 (70.6%) unique indication strings were mapped to 7844 disease terms. To effectively study the active ingredient, the drug names were further normalized into corresponding generic names according to RxNorm [23]. 392,639 out of 681,293 (57.6%) unique drug strings were mapped to 4717 generic drug names. The adverse reaction events in reaction files are coded in Medical Dictionary for Regulatory Activities (MedDRA) with in total 21,815 unique strings. After removing unknown words, the clean data files were linked together by case ID, resulting in 12,190,282 case reports, in which the prevalence of cases with multiple drugs, multiple diseases and multiple adverse events are 5,513,543, 7,950,105, 7,334,827 respectively, and the average numbers of drugs, diseases and adverse events in each case report are 2.91, 1.37 and 3.00 respectively.

#### Identifying OUD cohort by related terms

In original FAERS indication files, there are a few terms directly related to OUD, including “opium dependence”, “drug dependence of morphine type”, “opioid abuse”, and “opiate addiction”. The total number of individual reports with these specific OUD terms is only 11. To the identify potential OUD population, we took full advantage of intensive information in FAERS, in which we determined OUD patients based on whether opioid related drug names and drug use disorder related terms exist simultaneously in one individual patient. The drug use disorder was considered positive if either indication or reaction files includes related terms [24]. For example, if a certain patient had a drug record of “morphine” and had a reaction indicating “drug dependence”, then we built an indication record of OUD on this patient. This strategy results in total 57,293 potential OUD patients in this study.

#### Association rules mining on processed data

Association rules mining approach can observe frequent co-occurrence among multiple diseases. We applied frequent pattern-growth algorithm on indication dataset grouped by case report. The results may represent in the form of {X → Y}, for example, {fever, cancer → Covid-19} indicates when patients have fever and cancer, they are also likely to have Covid-19. Although the pattern is directed with an arrow, the directionality does not mean causation relationships but only indicates co-occurrences.

We evaluated with different combinations of parameters of frequent pattern growth algorithm in the previous study [11]. According to the performance in the previous experiment, the support in this study was set to no less than 0.000002, representing that the pattern at least appears in the ratio of 0.000002 from patients’ case reports; the maximum total number of diseases in each pattern was set to three; and lift was chosen as the measurement to mine the patterns, which represents the ratio of observed support to the expected support if X and Y are independent. With the minimum lift score of 1, a mined pattern{X → Y} has a degree to which the antecedent and consequent are dependent on each other and makes the rules reasonable.

$$lift\left(X\to Y\right)=\frac{support(X\vee Y)}{support(X)\times support(Y)}$$

#### Performance evaluation on mined OUD comorbidities

OUD comorbidities were defined as all the diseases presenting together with OUD in a rule. We used this definition in our previous studies of comorbidities of colorectal cancer [9] and Alzheimer’s disease [10]. We evaluated the performance of OUD comorbidities using a manually created a gold standard list from related literature reports. Precision and recall were reported as the performance measurement.

### Disease comorbidity network and network analysis

Association rules mining reports the connections between diseases based on chosen strength, while it cannot detect disease pairs with small sample size in database. We then used network-based approach to find and prioritize novel OUD comorbidities.

#### Constructing disease comorbidity network (DCN) based on mined rules

Given a pattern{X → Y}, all diseases in the set X and Y were collected with each disease representing a node, and each pair of disease was connected with an unweighted edge to construct the global network. For example, {OUD, cancer → fever, pain} will consist of 6 edges between diseases including {OUD, fever}, {OUD, pain}, {cancer, fever}, {cancer, pain}, {OUD, cancer}, {fever, pain}.

#### Prioritization of comorbidities associated with OUD

The random walk with restart (RWR) algorithm [25] was applied to rank the relevance for each disease with the seed, OUD. This algorithm can simulate a random walker from OUD and calculates the probability of each disease being a novel object. It repeatedly updated a probability vector P, in which we assume P_0_ is the initial vector, and P_k_ is the updated score vector at k_th_ step. M is the column-normalized adjacency matrix of disease comorbidities, and c is set to 0.15, which means the restart probability of seed. The loop terminated when ∣P_k+1_ – P_k_ ∣ < 1e−6, indicating the probability vector reaches a stable state.

$${P}_{k+1}=\left(1-c\right){M}^{T}{P}_{k}+c{P}_{0}$$

#### Evaluation on ranked results using decile method

The performance of ranked results at 10 deciles (top 0–10%, 10–20%, …, 90–100%) was evaluated using the list of manually curated known OUD comorbidities. The number of true positives in each decile was plotted in a histogram.

### Independent testing using patient EHR database

#### Database description

We applied a case–control statistical analysis using nation-wide and de-identified EHR data organized by IBM Watson Health Explorys. This retrospective population-level data represents 20% of US population from 317,000 providers and 360 hospitals across 50 states [26]. Explorys collected data from varieties of health information systems through a health data gateway (HDG) server with the data mapped into informatics ontologies. United Medical Language System (UMLS) is used for data normalization in searching and indexing [21]. Specifically, systematized nomenclature of medicine—clinical terms (SNOMED-CT) hierarchy is used to map findings, procedures, and diagnoses [27]; For the de-identification purpose, Explorys does not report the cohort population with size less than 10.

#### Study population

Patients were categorized based on their disease diagnoses drawn from Explorys. At the time of this study (Feb 10^th^, 2021), the Explorys database contains a sizeable study population of 74,574,090 patients including 370,470 who had a diagnosis of OUD, 3,739,290 with hypothyroidism, 499,290 with hyperthyroidism, and 5,051,290 with type 2-diabetes.

The odds of studied diseases (hypothyroidism, hyperthyroidism and type II diabetes) in patients with OUD compared with patients without OUD were calculated, adjusted for age, gender, race and corresponding diseases known risk factors. In the literature report, the risk factors of OUD includes mental disorders and history of substance abuse [28]. The risk factors of hypothyroidism in this study are hashimoto’s thyroiditis, operation on thyroid gland, radiation therapy [29]. Hyperthyroidism current risk factors include graves’ disease and chronic disease [30]. The type 2 diabetes clinical risk factors involve family history of diabetes mellitus, ethnicity, obesity and lifestyle factors such as lack of exercise, smoker [31]. The status of risk factors was determined on SNOMED-CT codes.

The exposure group contained patients who had any medical encounter with health care systems and had a diagnosis of OUD. The unexposed group contained patients who had a medical encounter but had no diagnosis of OUD. The outcome measurement was diagnosis of hypothyroidism, hyperthyroidism and type II diabetes separately.

### Statistical analysis

The EHR data in Explorys is de-identified in population-level rather than individual-level. Accordingly, we calculated odds ratios using Cochran-Mantel-Haernszel (CMH) method instead of performing regression analyses [14–16, 32, 33]. The adjusted odds ratio (AOR), 95% confidence interval, and *p* values (significance set at two-sided *p* values < 0.05) was calculated. We performed all the analyses with R, version 4.0.3.

## Results

### Association rules can capture known comorbidity diseases.

By applying Frequent Pattern (FP)-growth algorithm, we extracted in total 585,362 comorbidity association rules from indication data with minimum support as 0.000002 and minimum lift as 1. In total 111 diseases appeared together with OUD in these association rules. In the literature reports, comorbidities of OUD include psychiatric disorders such as anxiety, panic disorder, post-traumatic stress disorder (PTSD) and other substance dependence [2, 34]. Chronic Pain, HIV infection, and hepatitis c have also been confirmed as health problems related to OUD [35]. Based on the known comorbidity disease list we created, our OUD mined comorbidities achieved a precision of 0.38 and a recall of 0.78 separately.

### The network-based OUD ranking results demonstrate the robustness of DCN

Based on mined rules, we created an undirected and unweighted disease comorbidity network (DCN) with 1916 nodes and 32,175 edges. Using OUD as the seed, we prioritized diseases on the DCN in order to identify novel OUD comorbidities.

**Fig. 2:**
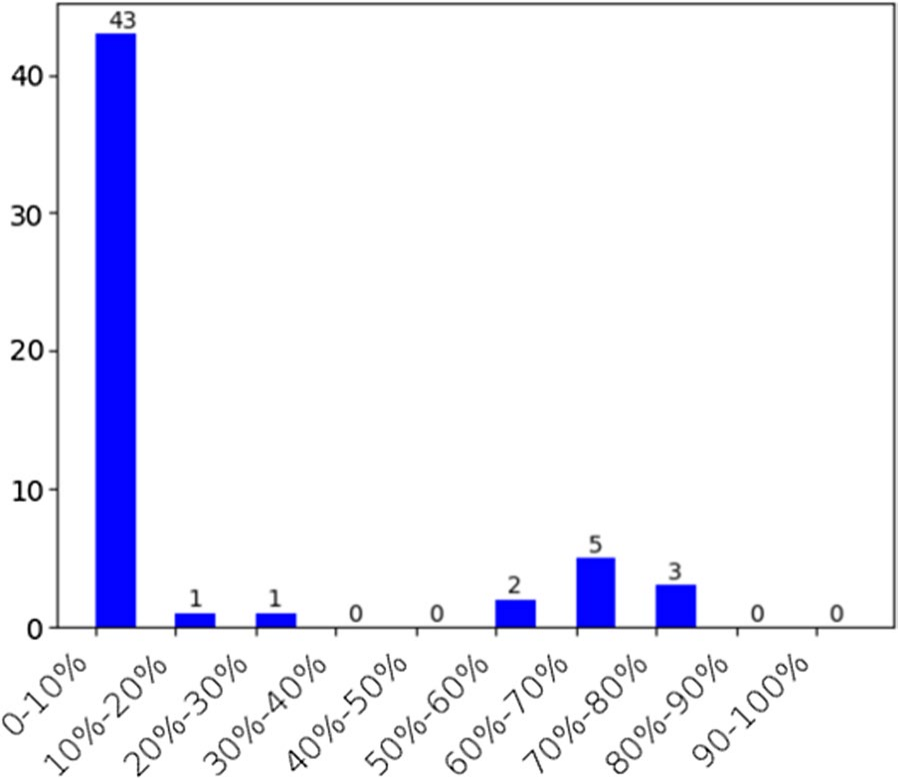
The numbers of true positives in 10 deciles of ranked diseases

Decile ranking was used to evaluate the performance of our ranked results. As shown in Fig. 2, 43 of 55 known OUD comorbidities were in the first decile with a precision of 0.78, which is higher than the precision of 0.38 for OUD comorbidities directly derived from mined association rules. These results demonstrated that network-based ranking effectively enriched OUD comorbidities among top.

### Hypothyroidism and diabetes mellitus were top-ranked unknown comorbidities

Table 1 shows the top-10 ranked diseases. Among the diseases that are not considered as comorbidities, hypertensive disease is common symptom of opioid withdrawal, constipation, nausea and gastroesophageal reflux disease are associated with side effects of opioid use. Hypothyroidism (relevance score: 0.00722648, ranked: 9) and diabetes mellitus (relevance score: 0.007102, ranked: 10) were prioritized with top scores but currently not well studied, which leads to our further investigation.

**Table 1:** Top 10 RWR-ranked diseases associated with OUD

	Disease	Score	Comorbidity
1	Hypertensive disease	0.013114	No
2	Pain	0.012274	Yes
3	Mental depression	0.011176	Yes
4	Anxiety disorders	0.010598	Yes
5	Gastroesophageal reflux disease	0.009415	No
6	Sleeplessness	0.008835	Yes
7	Constipation	0.008260	No
8	Nausea	0.007929	No
9	Hypothyroidism	0.007637	No
10	Diabetes mellitus	0.007473	No

### Patient demographics characteristics of the validation cohort

In the association mining results, hypothyroidism and diabetes present as top-rank comorbidities in OUD patients, which have not been demonstrated yet. To understand the impact of OUD on the thyroid gland, we were also interested in the potential connection of hyperthyroidism with OUD. The associations were further examined using statistical analysis from electronic health records. Demographics characteristics of the population we evaluated are shown in Table 2. The inconsistent summation is due to the fact that a patient can report multiple demographics(e.g., gender, race/ethnicity) at the same time.

**Table 2:** Demographics characteristics and outcomes

		All	OUD	Hypo	OUD + Hypo	Hyper	OUD + Hyper	Type2-Diabetes	OUD + Diabetes
Total		74,574,090	370,470	3,739,290	50,320	499,290	10,410	5,051,290	71,430
Sex	Female	40,008,930(54%)	188,490 (51%)	2,817,360(75%)	36,600 (73%)	382,990 (77%)	7,470 (72%)	2,590,240(51%)	37,770 (53%)
	Male	34,047,620(46%)	180,670 (49%)	905,130 (24%)	13,450 (27%)	114,460 (23%)	2,930 (28%)	2,452,870(49%)	33,420 (47%)
	Unknown	534,660 (1%)	1,410 (0%)	19,610 (1%)	290(1%)	2,060 (0%)	20(0%)	12,930 (0%)	290(0%)
Age	Adult	44,464,790(60%)	299,790 (81%)	1,656,970(44%)	30,650 (61%)	279,580 (56%)	7,060 (68%)	2,080,090(41%)	45,080 (63%)
	Senior	18,802,840(25%)	69,450 (19%)	2,020,550(54%)	19,740 (39%)	215,330 (43%)	3,370 (32%)	2,941,860(58%)	26,640 (37%)
	Junior	10,347,920(14%)	1,920 (1%)	23,830 (1%)	70(0%)	2,850 (1%)	10(0%)	13,680 (0%)	20(0%)
Race	White	40,641,550(54%)	289,930 (78%)	2,972,760(79%)	42,140 (84%)	352,120 (71%)	8,100 (78%)	3,446,160(68%)	50,940 (71%)
	African American	7,705,410(10%)	47,220 (13%)	257,260 (7%)	4,720 (9%)	77,830 (16%)	1,800 (17%)	843,830 (17%)	15,830 (22%)
	Asian	1,201,360(2%)	1,610 (0%)	58,210 (2%)	220(0%)	13,260 (3%)	60(1%)	101,440 (2%)	350(0%)
	Hispanic/ Latino	1,051,630(1%)	2,200 (1%)	31,630 (1%)	250(0%)	4,820 (1%)	60(1%)	70,900 (1%)	520(1%)
	Unknown	9,080,570(12%)	40,010 (11%)	490,390 (13%)	6,470 (13%)	63,960 (13%)	1,290 (12%)	630,950 (12%)	8,700 (12%)

By the time we accessed the database (Feb. 10th, 2021), there were in total 74,574,090 patients recorded in Explorys. Among the general population, 370,470 had a diagnosis of OUD, 3,739,290 had a diagnosis of hypothyroidism, 499,290 had a diagnosis of hyperthyroidism, and 5,051,290 had a diagnosis of type 2-diabetes. Among 370,470 patients with OUD, 50,320 had a diagnosis of hypothyroidism, 10,410 had a diagnosis of hyperthyroidism, and 71,430 had a diagnosis of type 2-diabetes.

### Positive associations between hypothyroidism, hyperthyroidism, type 2-diabetes and OUD

**Fig. 3:**
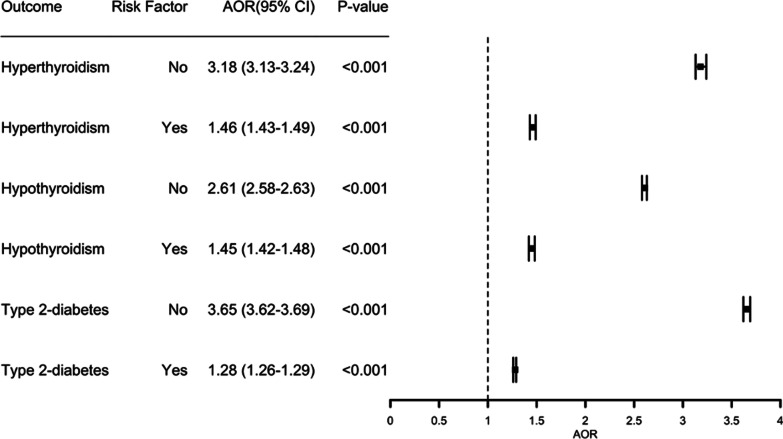
Associations of OUD with hyperthyroidism, hypothyroidism and type 2-diabetes. No—adjusted for demographics only, Yes—adjusted for both demographics and known risk factors

Figure 3 shows the results calculated using CMH method. Patients with diagnosis of OUD has higher odds of hyperthyroidism (AOR = 3.18, 95% CI 3.13–3.24, *p* value < 0.001), hypothyroidism (AOR = 2.61, 95% CI 2.58–2.63, *p* value < 0.001), type 2-diabetes (AOR = 3.65, 95% CI 3.62–3.69, *p* value < 0.001) than patients without a diagnosis of OUD, after controlling for age, gender, ethnicity.

After adjusting for additional confounders (risk factors for both OUD and the outcome), the odds of diagnosis of each outcome decreased but remained highly significant: hyperthyroidism (AOR = 1.46, 95% CI 1.43–1.49, *p* value < 0.001), hypothyroidism (AOR = 1.45, 95% CI 1.42–1.48, *p* value < 0.001), type 2-diabetes (AOR = 1.28, 95% CI 1.26–1.29, *p* value < 0.001).

## Discussion

In this study, we identified novel OUD comorbidities by mining the FAERS database of 12 million unique case reports and by network-based prioritization. Two top ranked novel OUD comorbidities (hypothyroidism, and type 2-diabetes) alone with hyperthyroidism were then independently validated using EHRs of 75 million unique patients.

Opioid may induce immunologic effects [34], however the association of OUD with how thyroid gland functions has not been widely studied. The association between hypothyroidism and depression has been discussed for years [36], and recent study shows that there is a moderate association of hypothyroidism with clinical depression [37]. Since OUD is highly related to depression [34], in this study, we investigated the association between hypothyroidism and OUD. Hypothyroidism is often overlooked due to its mild symptoms in the early stage, however, untreated hypothyroidism may lead to numbers of medical problems including obesity and heart disease [29]. Hyperthyroidism causes irregular heartbeat and accelerate the body’s metabolism resulting in weight loss [30]. Hypothyroidism and hyperthyroidism can be accurately identified by thyroid function tests, and treatments and medications are available with the evidence that most people respond well once the diseases have been diagnosed and treated [29, 30]. The positive association of OUD with both hyperthyroidism, hypothyroidism emphasizes the importance to recognize irregular thyroid and prevent delays in health care intervention among OUD population.

Type 2 diabetes has been recognized as one of the most concerned public health problems. Patients with type 2 diabetes have problems with producing enough insulin hormone to regulate sugar movement into cells [38]. The increased risk of diabetes in people with substance use has been verified, while most studies were conducted for alcohol [citation] and nicotine [39], and very few studies focused on the high prevalence of diabetes in OUD individuals. In animal studies, the possibility of diabetes associated with opioids has been demonstrated [6]. However, findings in humans remain inconclusive and controversial [6]. Our finding based on two independent large databases of patient health records provides strong evidence of positive association between OUD and type 2-diabetes in humans. The mechanisms underlying this strong positive association warrant further investigation. In our recent study, we showed that olanzapine, an anti-psychotic drug to treat schizophrenia (an OUD comorbidity), is associated with significantly higher OUD remission in patients with both OUD and schizophrenia [19]. Inspired by our finding, National Institute on Addiction (NIDA) is planning a prospective clinical trial of olanzapine in treating OUD. The strong positive association between OUD and type 2 diabetes suggests that controlling type 2 diabetes may prevent or mitigate OUD.

Due to limited usage of OUD-related terms in FAERS database, this study recognized OUD cohort not only using these terms but also identified the potential OUD population based on whether dependence-related terms or abuse-related terms and opioid-related drug simultaneously exist in one individual case. This approach probably introduces the false positives. For example, patients with alcohol addiction (a type of drug abuse), might take opioid medication to cure pain. And multiple drugs and drug use disorder keywords were mentioned in the same report, which may be tricky to assume the relationship between them. We will explore additional NLP methods that we used to identifying biomedical terms (Name Entity Recognition) as well as that we used to establish relationships (Relationship Extraction). Despite this limitation, our mined OUD disease comorbidity network is capable of identifying known disease co-occurrences with a precision of 38.7% and a recall of 78.2%. Patients take opioid medication to cure pain, therefore, it is understandable pain-related diseases are included in mined diseases. Opioid usage is often associated with side effects such as dizziness, sedation, and vomiting, which are related to the drug but are not necessarily included in gold standard comorbidity list. Some of OUD patients might experience opioid withdrawal with the symptoms including hypertension. These pain-related terms and side effect terms increased the false positive rate and they are reasonable in co-occurrence pairs. The low overall precision reflected that many of the mined OUD comorbidity associations were not in the evaluation dataset, many of which may be false negatives such as hypothyroidism-OUD. This is also the main goal of this two-prone study: identifying potential novel associations from FAERS and testing them using patient electronic records. Additionally, the associations of unknown diseases can be verified in Explorys, which would demonstrate mined DCN is able to discover novel comorbidities.

We used FAERS for potential OUD comorbidity discovery and the de-identified population-level Explorys EHR database for validation. FAERS was used for initial discovery but not for statistical validation, since it is crucial to adjust with the risk factors in statistical validation. However, FAERS does not have enough confounders to include—it is striking that a large amount of gender information is missing and no race information. Besides these important demographic characteristics, FAERS also lacks socioeconomic and lifestyle findings, such as insurance, patient’s family history of disease, exercise, and surgery information, which have a great impact on morbidities. Besides, for the variables included in FAERS, if patients do not have a certain disease in files, then we just assumed that this patient is in control group, while this individual might be a false negative since FAERS is a voluntary report system. And FAERS might also include the underdiagnosis, overdiagnosis, or misdiagnosis cases. Due to the huge data size, FAERS is favorable to provide us co-occurrence pairs from rough data mining. We used Explorys EHR database for hypothesis-driven validation, but not for initial discovery. The Explorys database contains population-level, not patient-level patient EHRs, which prevents any systematic hypothesis-free data-mining efforts. Recognizing both advantages and limitations of FAERS and Explorys EHR database, we designed this two-prone approach to identify novel OUD comorbidities using FAERS and then validate them using patient EHRs.

## Conclusions

Our study developed an integrated approach for identifying and validating novel OUD comorbidities from health records of 87 million unique patients (12 million for discovery and 75 million for validation), which can offer new opportunities for OUD mechanism understanding, drug discovery, and multi-component service delivery for co-occurring medical conditions among patients with OUD.

## Data Availability

The dataset for this article is available in the http://nlp.case.edu/public/data/oud_comorbidity. The code for this article is available in the https://github.com/misspanda95/OUD_comorbidities_study.

## Abbreviations

OUD: Opioid use disorder
DCN: Disease comorbidity NETWORK
EHRs: Electronic Health Records
FAERS: Food and Drug Administration Adverse Event Reporting System
FP: Frequent pattern
HDG: Health data gateway
HEAL: Help to end addiction
MDWG: Multidisciplinary Working Group
RWR: Random walk with restart
SNOMED-CT: Systematized Nomenclature of Medicine—Clinical Terms
UMLS: United Medical Language System
